# Biomarkers for the Evaluation of Pouch Inflammation: A Systematic Review

**DOI:** 10.1093/crocol/otac043

**Published:** 2022-11-24

**Authors:** Katherine Falloon, Benjamin L Cohen, Ronald Ottichilo, David Grove, Florian Rieder, Taha Qazi

**Affiliations:** Department of Gastroenterology, Hepatology & Nutrition, Digestive Diseases and Surgery Institute, Cleveland Clinic Foundation, Cleveland, OH, USA; Department of Gastroenterology, Hepatology & Nutrition, Digestive Diseases and Surgery Institute, Cleveland Clinic Foundation, Cleveland, OH, USA; Department of Inflammation and Immunity; Lerner Research Institute, Cleveland Clinic Foundation, Cleveland, OH, USA; Department of Inflammation and Immunity; Lerner Research Institute, Cleveland Clinic Foundation, Cleveland, OH, USA; Department of Gastroenterology, Hepatology & Nutrition, Digestive Diseases and Surgery Institute, Cleveland Clinic Foundation, Cleveland, OH, USA; Department of Inflammation and Immunity; Lerner Research Institute, Cleveland Clinic Foundation, Cleveland, OH, USA; Department of Gastroenterology, Hepatology & Nutrition, Digestive Diseases and Surgery Institute, Cleveland Clinic Foundation, Cleveland, OH, USA

**Keywords:** biomarker, ileal pouch-anal anastomosis, pouchitis, inflammatory bowel disease, ulcerative colitis

## Abstract

**Background:**

Ileal pouch inflammation is a common problem following ileal pouch-anal anastomosis (IPAA). Despite its prevalence, diagnosis remains multimodal and requires endoscopy. The use of biomarkers in the prediction of and/or association with pouchitis has not been well characterized. We performed a systematic review to summarize the available evidence.

**Method:**

A search of Ovid, MEDLINE, Cochrane Library, EMBASE, and Web of Science was conducted. Inclusion criteria included studies evaluating biomarkers for the evaluation and prediction of inflammation in patients with IPAA utilizing pouchoscopy as the gold standard. Exclusion criteria included studies on the role of the microbiome or genetic markers.

**Results:**

A total of 28 studies, 5 case-control studies, and 23 observational cohort studies were identified. Fecal biomarkers were assessed in 23 studies, of which fecal calprotectin was the most commonly studied with sensitivities ranging from 57% to 92% and specificities from 19% to 92%. Six studies examined serum biomarkers. None of the serum biomarkers demonstrated a high sensitivity or specificity in association with pouch inflammation. Six studies described the longitudinal assessment of biomarkers. Of these studies, only three reported a predictive role of biomarkers in diagnosing endoscopic inflammation.

**Conclusions:**

Biomarkers have emerged as a potential option to improve the management of pouchitis given the relative ease of sampling compared to pouchoscopy. Unfortunately, the evaluated biomarkers have not consistently demonstrated accuracy in predicting inflammation. Moreover, these biomarkers have not been reliably shown to be sensitive or specific in association with endoscopic pouch inflammation to merit their widespread use in clinical practice.

## Teaser

Although biomarkers offer a noninvasive approach to the evaluation of ileal pouch inflammation, a systematic review of the literature found that no biomarker has consistently and reliably demonstrated accuracy in predicting or diagnosing pouch inflammation.

## Graphical Abstract

**Graphical Abstract:**
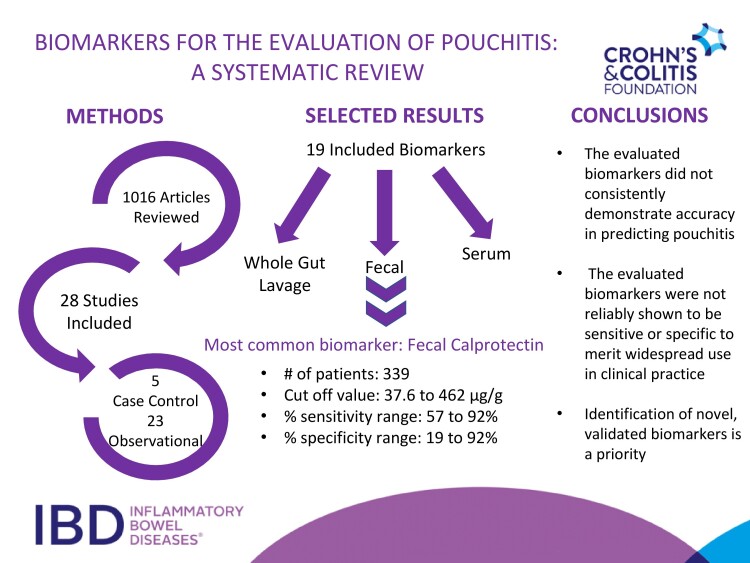

## Introduction

Nearly 30% of patients with ulcerative colitis (UC) eventually require surgery for medically refractory colitis, dysplasia, or other complications of inflammatory bowel disease. Ileal pouch-anal anastomosis (IPAA) has become the main surgical approach of choice following total proctocolectomy in UC.^1^ Following IPAA creation, inflammation of the pouch occurs in nearly 60% of patients, manifesting as acute pouchitis, chronic pouchitis, or Crohn’s-like diseases of the pouch.^2,3^ Classic symptoms of pouch inflammation include diarrhea, abdominal pain, and frequency, but these can also occur from alternative etiologies. An accurate means of diagnosing pouchitis and differentiating it from non-inflammatory pouch disorders in symptomatic patients is thus of vital importance. In addition, routine early detection of evolving pouchitis is an unmet need that may allow for early intervention.

The current gold standard for the objective assessment of the pouch is pouch endoscopy, also termed pouchoscopy, which is limited not only by its invasive nature but also by its cost.^4^ Several surrogate markers have been commonly used for the assessment of pouch inflammation in clinical practice. Despite their utilization, no biomarkers have been validated for evaluation or prediction of pouch inflammation and most biomarkers have not been tested against a thorough gold standard or undergone longitudinal testing in the same patient over time.

The purpose of this systematic review is to summarize the available literature on biomarkers for the prediction, association, and longitudinal assessment of pouch inflammation as tested against the gold standard of pouchoscopy. In this work, we focus on fecal and serum biomarkers, not on genetic or microbiome evaluation given the inherent limitations associated with the utilization of these modalities as objective predictors of active inflammation.

## Material and Methods

### Search Strategy

This systematic review was conducted in accordance with the Preferred Reporting Items for Systematic Review and Meta-Analyses guidelines. Studies were identified by searching Ovid, MEDLINE, Cochrane Library, EMBASE, and Web of Science from inception to February 18, 2022. Each database was searched using medical subject heading (MeSH) and keywords and synonyms derived from them, including biomarkers, IPAA, pouchitis, diagnosis, and prognosis. A detailed description of the search strategies for each of the databases is listed in Supplementary Tables 1–3. The identified studies were first screened in abstract form, with the removal of duplicates and the exclusion of abstracts outside of eligibility criteria. Full-text articles were then reviewed to identify those meeting inclusion criteria. All references of full-text articles were also reviewed manually to identify any additional relevant publications. The final identified studies were then used for data extraction (Supplementary Table 4).

### Inclusions and Exclusion Criteria

Eligibility criteria were set *a priori* by the study authors. Inclusion criteria were original studies with the following study designs: randomized or non-randomized observational cohort studies, case-control studies, or case series involving at least 10 patients. Other inclusion criteria were patients with an IPAA (including J, W, and S pouches) regardless of pre-operative diagnosis; age 18 years or older; utilization of easily attainable clinical biomarkers obtained from serum, stool, urine, or breath for the evaluation and/or prediction of pouch inflammation; and comparison of the biomarker against the gold standard of pouch endoscopy. Exclusion criteria were narrative reviews or editorials; languages other than English; studies with fewer than 10 patients; studies investigating only microbiome and/or genetic markers; and patients with a diverting ileostomy, Kock Pouch, or alternative enteric-colonic anastomoses.

### Outcomes of Interest

The outcomes of interest were the comparison of the biomarker to inflammation status as assessed via pouchoscopy using either correlative statistics or sensitivity and specificity, prediction of future episodes pouchitis via biomarkers, and the longitudinal evaluation of pouchitis using biomarkers.

### Study Selection and Data Extraction

Two reviewers (KF and TQ) independently screened all titles and abstracts identified and selected studies for inclusion based on eligibility criteria. Disagreements regarding eligibility for inclusion were resolved through consensus with a third reviewer (FR). The following variables were extracted from each of the studies: first author name, journal, year of publication, study design, number of patients, age of patients, pre-operative history of patients, biomarker evaluated, biomarker cutoff, comparative endoscopic criterion, and sensitivity and specificity data and/or correlative statistics.

### Risk of Bias in Individual Studies

KF and TQ independently assessed all non-randomized studies for risk of bias using the Newcastle-Ottawa Scale.^5^ Any discrepancies in scoring were resolved via discussion among the two reviewers with final consensus by a third reviewer (FR; Supplementary Table 5).

## Results

A total of 1016 studies were screened in abstract form, and upon removal of 106 duplicates, 910 studies remained. Of these, 768 were excluded due to not meeting eligibility criteria. About 124 studies were then assessed in full-text format with 95 ultimately excluded (32 were only abstracts, 9 were in the incorrect patient population, 16 had the incorrect study design, 19 presented a non-eligible outcome, 13 did not report a biomarker, and 6 did not compare a biomarker to a gold standard). A total of 28 studies were included in the final qualitative analysis. Of these, 23 were observational cohort studies and 5 were case-control studies. The PRISMA diagram is shown in Supplementary Figure 1.

Serum biomarkers were evaluated in 6 studies and fecal biomarkers, with or without serum biomarkers, were assessed in 23 studies. Four studies examined the use of whole gut lavage (WGLF), in which patients complete a bowel preparation, and proteins in the stool are measured following clearance of the fecal effluent.

About 25 studies assessed the role of a biomarker in association with pouch inflammation. Three studies evaluated biomarkers as predictors of subsequent pouch inflammation. Six studies also reported longitudinal assessments of biomarkers in relation to pouch inflammation.

### Prediction of Pouch Inflammation

Of the 28 compiled studies, three studies evaluated the role of biomarkers in the prediction of inflammation following IPAA creation.^6–8^ The details of these studies are summarized in Table 1. The examined biomarkers include fecal calprotectin, fecal lactoferrin, and the neutrophil–lymphocyte ratio. Fecal calprotectin is a stable protein resistant to degradation found in the cytosol of neutrophils and certain macrophages that is released into the lumen of the GI tract in the setting of active inflammation.^9^ It has been validated as a marker of luminal inflammation but its association with pouchitis remains unclear. Lactoferrin is a protein released from active polymorphonuclear leukocytes and can be found in the stool when there is active inflammation in the gastrointestinal tract. It is believed to act against enteric pathogens.^10–12^

**Table 1.** Prediction of stool biomarkers.

Author	Year	Study design	Patient #	Median age (range)	Pre-Op diagnosis	Pouchitis definition	Biomarker	Cutoff (µg/g)	Method; assay	Association between biomarker and PDAI	Sensitivity (%)	Specificity (%)
Yamamoto	2015	Case control	60	34 (21–64)	UC	PDAI ≥ 7	Fecal calprotectin	56	ELISA; Human Calprotectin Kit 9, (Cell Sciences, Canton, MA, USA)	*Endoscopy subscore*: *r* = 0.696 *p* < 0.0001 *Total PDAI*: *r* = 0.626 *p* < 0.0001	100	84
Yamamoto	2015	Case control	60	34 (21–64)	UC	PDAI ≥ 7	Fecal lactoferrin	50	ELISA; Human Calprotectin Kit 9, (Cell Sciences, Canton, MA, USA)	*Endoscopy subscore*: *r* = 0.676 *p* < 0.0001 *Total PDAI*: *r* = 0.582 *p* < 0.0001	90	86
Machiels	2017	Prospective cohort	19	45 (35–51)	UC	Symptoms + endoscopic inflammation	Fecal calprotectin	315.5	QI; Quantum Blue Calprotectin High range Assay, (Buhlmann Laboratories AG Schonenbuch, Switzerland)	AUC = 0.833	83.3	72.7
Nishida	2020	Retrospective cohort	49	44 (33–54)	UC	mPDAI ≥ 5	Neutrophil lymphocyte ratio	2.15	XE-500 hematology analyzer; (Sysmex, Kobe, Japan)	AUC = 0.68	72.2	67.7

Abbreviations: AUC: area under the curve, ELISA: enzyme linked immunosorbent assay; PDAI: pouchitis disease activity index; pre-op: pre-operative; QI: quantitative immunochromatography; UC: ulcerative colitis;

The largest study was a case-control study performed by Yamamoto et al. which followed 60 patients who underwent ileostomy closure and IPAA creation.^6^ Stool samples assessing fecal calprotectin and fecal lactoferrin were obtained every two months for up to 12 months following ileostomy closure. Pouchoscopy was performed in all patients with either development of symptoms or at 12 months. Within the study period, 10 patients developed pouchitis, diagnosed as a pouchitis disease activity index (PDAI) ≥ 7 (median: 11, range: 8–14). The time from ileostomy closure to the diagnosis of pouchitis ranged from 6 to 12 months, with a median of 10 months. The resultant sensitivitiy and specificitiy were 100% and 84% for fecal calprotectin and 90% and 86% for fecal lactoferrin, as summarized in Table 1. Of note, the authors also measured C-reactive protein (CRP), which remained at a normal level and did not change significantly throughout the study duration in both patients with and without pouchitis.^6^

In a cohort of 19 patients who underwent total proctocolectomy and IPAA creation, Machiels et al. performed serial assessments of fecal calprotectin along with endoscopic evaluations at one, 3, 6, and 12 months following ileostomy closure. Over this follow-up period, eight patients developed pouchitis, defined as symptoms of pouchitis along with the presence of endoscopic inflammation. Sensitivity and specificity in predicting pouchitis were slightly inferior to that found by Yamamoto et al. (83% and 73%). However, Machiels et al. did go on to perform a receiver operating characteristic (ROC) analysis of fecal calprotectin at one month following ileostomy closure, which demonstrated a robust area under the curve (AUC) of 0.833 (Table 1). Concentrations of stool short-chain fatty acids were also evaluated in the study and were not found to be associated with the future development of pouchitis.^7^

Finally, Nishida et al. evaluated the role of a neutrophil–lymphocyte ratio as a predictor of the subsequent development of pouchitis, diagnosed via a modified pouchitis disease index score (mPDAI) greater than or equal to 5.^8^ The authors included 49 patients, of whom 18 developed pouchitis over a median follow up of 18 months. The ROC analysis demonstrated that the best cutoff value for the neutrophil–lymphocyte ratio was a value greater than or equal to 2.15. This value corresponded to an AUC of 0.68 with the lowest reported sensitivity (68%) and specificity (72%) in predicting the subsequent development of pouchitis following stoma closure (Table 1).

### Association with Pouch Inflammation

 A total of 25 out of the 28 studies describe an association of serum and stool biomarkers with pouch inflammation. All 25 are cross-sectional studies with samples taken at or close to the time of pouchoscopy. The details of these studies are compiled in Tables 2–4 and Supplementary Tables 6 and 7.

**Table 2.** Association of selected serum markers with pouch inflammation.

Author	Year	Study design	Patient #	Median age (range)	Pre-Op diagnosis	Pouchitis definition	Biomarker	Association between biomarker and PDAI	Sensitivity (%)	Specificity (%)
Lu	2010	Prospective cohort	83	43 (29–57)	UC	Endoscopic PDAI ≥ 2	CRP > 0.7 g/dL	*Endoscopy subscore:* *r* = 0.28 *p* = 0.06 AUC pouch body inflammation = 0.63 *Total PDAI*: NP	69.7	63.6
Matalon	2015	Prospective cohort	71	44 (35–52)	UC	PDAI ≥ 7	CRP	*Endoscopy subscore*: NP *Total PDAI*: *r*= 0.584 *p*= 0.001	NP	NP
Matalon	2015	Prospective cohort	71	44 (35–52)	UC	PDAI ≥ 7	Serum A1AT >189 mg/dL	*Endoscopy subscore*: NP *Total PDAI*: *r*= 0.583 *p*< 0.001	55.6	100
Wang	2013	Prospective cohort	185	46 (29–61)	UC (163) CD (3) Indeterminate (18)	mPDAI ≥ 5	Serum serotonin	*Endoscopy subscore*: OR 1.8, 95% CI 1.2–2.8 *p*< 0.05 *Total PDAI:* No correlation	NP	NP
Sandborn	1995	Prospective cohort	52	36–64 (26–77)	UC (47) FAP (5)	PDAI ≥ 7	p-ANCA	*Endoscopy subscore: *Correlation to all findings except ulceration *Total PDAI*: *r*= 0.39 *p*< 0.01	NP	NP
Yasuda	1998	Case control	49	32–37 (22–56)	UC	Pouchitis by St. Mark’s criteria	p-ANCA	No correlation	NP	NP
Werner	2013	Case control	41	19–26 (11–49)	UC (36) FAP (5)	PDAI ≥ 7	Serum anti-GP-2	Significantly higher level compared to controls (*p*< 0.05)	NP	NP

Abbreviations: A1AT: alpha 1 anti-trypsin; AUC: area under the curve; CD: Crohn’s disease; CI: confidence interval; CRP: C-reactive protein; FAP: familial adenomatous polyposis; GP-2: glycoprotein 2; IgG: immunoglobulin G; OR: odds ratio; p-ANCA: perinuclear anti-neutrophil cytoplasmic antibody; PDAI: pouchitis disease activity index; pre-op: pre-operative; UC: ulcerative colitis

#### Serum markers associated with pouch inflammation

Six studies examined the association of serum biomarkers with pouch inflammation. The serum markers include CRP, Alpha-1-Antitrypsin (A1AT), serotonin (5-HT), perinuclear anti-neutrophil cytoplasmic antibody (pANCA), and serum immunoglobulin glycoprotein 2 (GP2).

##### C-reactive protein:

CRP is an acute phase reactant synthesized by the liver. In the setting of inflammation, hepatocyte activation leads to an increase in CRP that can be detected in the circulation.^13^ CRP is widely used as a surrogate marker for inflammation. In patients with IBD, CRP has been studied as a marker for detecting or predicting luminal inflammation and response to therapy.^14^

Only one study by Lu et al.is dedicated solely to the clinical utility of CRP in patients with IPAA.^15^ In this study, 83 patients with IPAA had a serum CRP obtained within 2 weeks of endoscopic evaluation. Numerically higher CRP levels were seen in patients with chronic inflammatory conditions, specifically chronic pouchitis, Crohn’s disease-like phenotype of the pouch, and surgical complications of the pouch, not defined in the study but reported as significant inflammation occurring within 12 months of ileostomy takedown. However, there was no significant difference in the levels of CRP among different pouch diagnoses, leading the authors to conclude that the level of CRP elevation was not useful in differentiating pouch conditions. As outlined in Table 2, while there was a correlation between CRP and the endoscopic appearance of the pouch regardless of diagnosis, across multiple sub-analyses the AUC reported for pouch body inflammation was 0.63. Sensitivity and specificity remained low at 70% and 64%, respectively.

Nine other studies have compared CRP to other biomarkers for the evaluation of pouch inflammation with mixed results (Table 2). The aforementioned study by Yamamoto et al. reported no change in CRP, which remained at normal levels throughout the study period of 12 months in all patients.^6^ Matalon et al. obtained CRP testing in 71 patients with IPAA.^16^ Median CRP levels were significantly higher in patients with a PDAI ≥ 7 at 9.07 (2.35–23.9) mg/dL versus 4.37 (2.12–7.9) mg/dL for patients with a PDAI of <7. Moreover, the authors reported a significant correlation between CRP and PDAI (0.584).^17^ Unfortunately, sensitivity, specificity, and AUC were not reported. Farkas et al. found higher CRP levels in 17 patients with pouchitis, but there was no correlation with other biomarkers. Pakarinen et al., on the other hand, noted similar CRP levels in 15 patients with elevated fecal calprotectin compared to 17 with normal calprotectin levels.^18^ In a recent study by Ollech et al., CRP was compared to fecal calprotectin as a marker for pouch inflammation in a cohort of 156 patients with IPAA.^19^ The authors found that median CRP values were higher in patients with pouchitis compared to patients without pouchitis. However, the accuracy of CRP to identify pouchitis was lower than that of fecal calprotectin, with an AUC of 0.59 compared to an AUC of 0.68 and no sensitivity or specificity was reported. Importantly, the authors also reported that the CRP levels increased with higher endoscopic scores but not higher histologic subscores. Finally, four other studies did not provide objective data but did state there was no correlation of CRP to pouchitis or to other evaluated biomarkers.^20–23^

##### Alpha-1 antitrypsin:

A1AT is an acute phase reactant produced by hepatocytes as well as inflammatory cells.^24^ This serine protease inhibitor acts to mitigate tissue injury induced by active inflammation.

In the previously described cohort of 71 patients, Matalon et al. characterized serum A1AT for the evaluation of pouchitis.^25^ Patients with a PDAI ≥ 7 had a significantly higher serum A1AT (183.0 mg/dL) compared to patients with a PDAI < 7 (167.7mg/dL). AUC was not calculated, however, a correlation between PDAI score and serum A1AT level (*r* = 0.583) was noted. With an A1AT cutoff of 189 mg/dL, the authors reported a sensitivity of 56%, specificity of 100%, and a positive predictive value of 100% to yield a PDAI ≥ 7 (Table 2). However, the degree of A1AT elevation could not be used to distinguish acute/recurrent pouchitis from chronic pouchitis.

##### Serotonin:

The largest producers of 5-HT in the body are enterochromaffin cells in the gut, and the role of 5-HT in the pathophysiology of IBD has been suggested in studies describing a down-regulation of re-uptake transporters in patients with IBD compared to healthy controls.^26^ Additional studies have also described elevated 5-HT in Crohn’s disease patients with active disease compared to remissive disease.^27^

In a population of 185 patients, Wang et al. obtained serum serotonin levels prior to pouch endoscopy to evaluate the role of serotonin in association with pouch inflammation (Table 2).^28^ Serum 5-HT did not differ among patients with normal pouches, irritable pouch, inflammatory pouch disorders, and surgical complications of the pouch. Serotonin levels did significantly correlate with PDAI endoscopy subscore (OR = 1.9, 95% CI: 1.2–2.9) and total endoscopy score (OR = 1.8; 95% CI: 1.2–2.8), however, there was no correlation between serum 5-HT and symptoms as assessed through the PDAI. Sensitivity, specificity, and AUC were not reported.

##### Perinuclear anti-neutrophil cytoplasmic antibody:

Several studies have suggested that p-ANCA is present in patients with UC but does not correlate with disease extent, activity, or duration.^29–31^

The presence of p-ANCA has been examined in two studies in patients with IPAA (Table 2).^32,33^ In the initial study by Sandborn et al., the presence of pANCA was studied in a cohort of 52 patients, including 19 patients with a prior history of UC and an IPAA with pouchitis, 18 with a prior history of UC and an IPAA without pouchitis, 5 with FAP and an IPAA, and 10 with a Brooke ileostomy.^32^ The authors report significant positive correlations between pouchitis disease activity as measured by the PDAI score and pANCA levels and titers (*r* = 0.39 and *r* = 0.35, respectively). There were also significant correlations between pANCA levels and titers and individual components of the PDAI, including the endoscopic findings of edema, granularity, friability, loss of vascularity, and the presence of a mucous exudate. However, endoscopic ulceration did not correlate with pANCA levels. Sensitivity, specificity, and AUC were not reported.

A subsequent study investigated the presence of pANCA in a cohort of 49 patients, of whom 25 had acute pouchitis as defined via St. Marks Criteria, including clinical symptoms of diarrhea as well as endoscopic and histologic features of acute inflammation. The other 24 patients had a normal pouch. No significant difference was noted in p-ANCA positivity between patients with and without pouchitis using both immunofluorescence and ELISA techniques, and sensitivity and specificity were not reported (Table 2).

##### Glycoprotein 2:

Pancreatic auto-antibodies have been found to be more prevalent in patients with Crohn’s disease, specifically early-onset Crohn’s disease, ileocolonic disease, and a penetrating phenotype.^34,35^ The antigenic target has been identified as anti-glycoprotein 2 (anti-GP2).^36^

Serum anti-glycoprotein antibodies were assessed in one study of 42 patients with IPAA (Table 2).^21^ The cohort included 13 patients with recurrent, acute pouchitis, encompassing patients with episodes of antibiotic-responsive pouchitis with repeated flares up to four times per year, 13 with chronic pouchitis, defined as patients requiring antibiotic or anti-inflammatory therapy for at least 4 weeks, or patients having more than five flares per year, 10 with a normal pouch, and five with FAP. The serum levels of anti-GP2 antibodies of both immunoglobulin g (IgG) and IgA were significantly higher in patients with chronic and recurrent acute pouchitis than in patients with normal pouches, regardless of pre-operative diagnosis. The authors also compared the levels of anti-GP2 antibodies in pouch patients to patients with UC (*n* = 7) and Crohn’s disease (*n* = 7) and reported that sera levels of anti-GP2 were similar, suggesting a similar pathophysiology in both conditions. Once again, sensitivity, specificity, and AUC were not reported.

### Stool Markers Associated with Pouch Inflammation

Stool biomarkers have been investigated as biomarkers for pouchitis with the hope that, while more onerous to collect than a serum sample, they might prove more reliable. Unfortunately, interpretation of this data is limited due to small sample size (ranging from 19 to 156 patients) and/or differing proposed cut-offs with correspondingly varied sensitivities and specificities.

#### Fecal calprotectin:

As described above, fecal calprotectin is released into the luminal GI tract in the setting of active inflammation. Six studies comprising a total of 339 patients have been conducted with cutoff values ranging from 37.6 to 462 µg/g with corresponding sensitivities and specificities of 57% to 92% and 19% to 92%, respectively (Table 3). AUCs were calculated in two studies (0.78, 0.832).^37,38^

**Table 3.** Association of fecal calprotectin with pouch inflammation.

Author	Year	Study design	Patient #	Median age (range)	Pre-op diagnosis	Pouchitis definition	Biomarker	CutOff (µg/g)	Method; assay	Association between biomarker and PDAI	Sensitivity (%)	Specificity (%)
Thomas	2000	Prospective cohort	24	NP	UC (16) FAP (8)	Macroscopic inflammation and histologic inflammation	Fcalpro	NP	ELISA; NP	All patients with inflammation had elevated fcalpro	NP	NP
Pronio	2016	Prospective cohort	40	52 (33–71)	UC	PDAI ≥ 7	Fcalpro	66.2 37.6	ELISA; Calprest Test (Eurospital, Trieste, Italy)	*Endoscopy subscore: *NP *Total PDAI*: *r* = 0.55 *p* = 0.002 AUC: 0.832	85 92	38 19
Johnson	2009	Prospective cohort	54	47	UC (46) FAP (8)	PDAI ≥ 7	Fcalpro	92.5	ELISA; PhilCal Test (Calpro, Oslo, Norway)	*Endoscopy subscore: * *r* = 0.605 *p*≤ 0.0001 *Total PDAI:r* = 0.71 *p* ≤ 0.001	90	76.5
Farkas	2015	Prospective cohort	33	30–40	UC	PDAI ≥ 7	Fcalpro	262	ELISA; (Quantum Blue, Buhlmann Laboratories Ltd, Schonenbuch)	*Endoscopy Subscore:* Significant association w *p* = 0.0001 *Total PDAI:* AUC = 0.78	67	89
Pakarinen	2010	Prospective cohort	32	24 (17–31)	UC	Histologic neutrophil count + episodes of pouchitis	Fcalpro	300	ELISA; PhilCal Test (Calpro, Oslo, Norway)	Histologic neutrophil count *r* = 0.715 *p* < 0.001 Episodes of pouchitis *r* = 0.457 *p* < 0.01	57	92
Ollech	2021	Prospective cohort	156	43 (35–58)	UC	Endoscopic PDAI ≥ 5	Fcalpro	462	ELISA; NP	Severity of pouchitis *r* = 0.526 *p* = 0.0017	66.7	82.4

Abbreviations: AUC: area under the curve; ELISA: enzyme linked immunosorbent assay; FAP: familial adenomatous polyposis; Fcalpro: fecal calprotectin; NP: not provided; PDAI: pouchitis disease activity index; pre-op: pre-operative; UC: ulcerative colitis

The role of fecal calprotectin in diagnosing pouch inflammation was first examined by Thomas et al.^39^ The authors performed 24-hour stool collections in 24 patients with IPAA and found that both endoscopic and histologic evidence of inflammation correlated with stool calprotectin concentrations. Pronio et al. then prospectively studied 40 patients with an IPAA and found at a cutoff of 66.2 µg/g, fecal calprotectin demonstrated a sensitivity of 85% and specificity of 38% with an AUC of 0.832 in detecting pouchitis, defined as a PDAI ≥ 7. Similar prospective cohort studies by Johnson et al. in 54 patients with IPAA and Farkas et al. in 33 patients once again demonstrated an association of fecal calprotectin with pouch inflammation The AUC was not provided in the Johnson study but was reported as 0.78 by Farkas et al. (Table 3).^,13,40^

In a prospective study of 32 patients with IPAA, Pakarinen et al. elected a different approach.^18^ Instead of correlating fecal calprotectin to PDAI score, the authors evaluated the correlation of fecal calprotectin to histologic neutrophil count in the distal ileum (*r* = 0.0175, *p* < 0.001) and to the maximum daily frequency of bowel movements (*r* = 0.610, *p* < 0.001). They also noted that mean fecal calprotectin level increased with the number of episodes of pouchitis, with a cutoff of 300 µg/g found to have a sensitivity of 57% and specificity of 92% in the detection of recurrent pouchitis, defined as the presence of recurrent pouch inflammation at least four times in one year (Table 3).

The most recent study of fecal calprotectin was conducted by Ollech et al. in 156 patients with a prior history of UC and IPAA. In this study, the authors sought to assess the association of fecal calprotectin with the endoscopic subscore of the PDAI.^19^ Notably, patients with a prior history of FAP were excluded from the study. The authors reported that fecal calprotectin levels were significantly higher in patients with pouchitis compared to patients without pouchitis. There was no significant difference in the levels of fecal calprotectin among patients with different pouch phenotypes (normal pouch, recurrent acute pouchitis, chronic pouchitis, Crohn’s-like disease of the pouch) when stratified according to the presence or absence of pouchitis (PDAI above or below 7). Sensitivity and specificity at various fecal calprotectin cut-offs are summarized in Table 3.

#### Fecal lactoferrin:

As described above, fecal lactoferrin is also found in the stool when there is active inflammation in the GI tract. Four studies encompassing 209 patients investigated the role of fecal lactoferrin in association with pouch inflammation. The details of these studies are reported in Table 4.

**Table 4.** Association of fecal lactoferrin with pouch inflammation.

Author	Year	Study design	Patient #	Median age (range)	Pre-op diagnosis	Pouchitis definition	Biomarker	CutOff (µg/g)	Method; Assay	Association between biomarker and PDAI	Sensitivity (%)	Specificity (%)
Scarpa	2011	Prospective cohort	32	50 (43–58)	UC	PDAI ≥ 7	Fecal lactoferrin	<7	ELISA; IBD-SCAN (TechLab, Blacksburg, VA)	Correlated with mucosal ulcers and histologic pouchitis	NP	NP
Parsi	2004	Prospective cohort	60	42–53 (36–57)	UC	PDAI ≥ 7	Fecal lactoferrin	7 13	NP	*Endoscopy subscore*: *r* = 0.73 *p* < 0.001 *Total PDAI*: *r* = 0.73 *p* = 0.001 AUC = 0.992	100 97	85 92
Lim	2008	Prospective cohort	32	47 (40–53)	UC	PDAI ≥ 7	Fecal lactoferrin	≥7.25	ELISA; IBD-EZ VUE (TechLab, Blacksburg, VA)	NP	100	82
Gonsalves	2013	Prospective cohort	85	42 (36–49)	UC	PDAI ≥ 7	Fecal lactoferrin	≥7.25	ELISA; IBD EZ VUE. (TechLab, Blacksburg, VA)	NP	100	92

Abbreviations:: AUC: area under the curve; ELISA: enzyme linked immunosorbent assay; IBD: inflammatory bowel disease; NP: not provided; PDAI: pouchitis disease activity index; pre-op: pre-operative; UC: ulcerative colitis

Scarpa et al. evaluated fecal lactoferrin in 32 consecutive patients with IPAA.^23^ Ten patients included in the study had a clinical diagnosis of pouchitis, defined as a PDAI ≥ 7. Although the authors reported that fecal lactoferrin correlated directly with the presence of mucosal ulcers, immune cell infiltration, and the histologic diagnosis of pouchitis, correlation coefficients, and sensitivities and specificities were not provided. Parsi et al. investigated the role of fecal lactoferrin in a prospective cross-sectional study of 60 patients with IPAA. Though unable to distinguish between different types of pouch inflammation (eg cuffitis versus Crohn’s-like disease of the pouch), at a cutoff of 13 µg/mL fecal lactoferrin was able to distinguish patients with irritable pouch syndrome from those with true inflammation with a sensitivity of 97% and a specificity of 92% (at a cuff-off of 7 µg/mL, these values changed to 100% and 85%, respectively).^41^ Similar findings were obtained by Lim et al. in a smaller prospective study of 32 patients, 11 with pouchitis (defined as a PDAI ≥ 7) and 21 without pouchitis (Table 4).^42^ The largest prospective study was conducted by Gonsalves et al. with a sample size of 85 patients.^43^ The cohort included 24 patients with pouchitis, defined as a PDAI ≥ 7, and 61 patients without pouchitis. The authors found a sensitivity of 100% and specificity of 92% to diagnose pouchitis.

#### Fecal alpha-1 anti-trypsin:

A1AT is not only produced by hepatocytes but also by intestinal epithelial cells.^44^ Fecal A1AT has been used as a sensitive marker for the evaluation of protein-losing enteropathy and has also been used for the evaluation of IBD.^45,46^ Fecal A1AT has been examined for the evaluation of inflammation of the pouch in two studies.

A study by Boerr et al. looked at fecal A1AT in 28 patients with IPAA and five with an ileostomy (Supplementary Table 6).^47^ Of the patients with an IPAA, 11 had active pouchitis with a PDAI ≥7, and 17 either had pouchitis in remission or had never had pouchitis. The authors found that with a cutoff of 20.5 mg fecal A1AT had a sensitivity of 80% and specificity of 97% in identifying pouchitis. There was a significant positive correlation observed between PDAI and fecal A1AT (*r* = 0.702), but there were weaker correlations with fecal A1AT and clinical, endoscopic, and histologic sub-scores of the PDAI. The aforementioned study by Parsi et al. also reported the results of fecal A1AT in 49 patients.^41^ Twenty-five patients were in the inflammatory pouch group, with a PDAI ≥ 7, cuffitis, or Crohn’s disease, 11 had irritable pouch syndrome, and 13 were asymptomatic. The ROC reported by the authors showed that fecal A1AT was unable to distinguish patients with irritable pouch syndrome from those with inflammation of the pouch. A1AT did not correlate with PDAI or individual sub-scores of the PDAI.

#### Exploratory stool markers

Matrix metalloproteases (MMP) have also emerged as potential biomarkers (Supplementary Table 6). Stallmach et al. found a statistically significant increase in levels of MMP-1 and MMP-2 in 11 patients with pouchitis and a prior history of UC, defined as a PDAI ≥ 7, compared to 22 patients without pouchitis, including 14 patients with a prior diagnosis of UC and 8 patients with FAP.^48^ Farkas et al. evaluated the role of fecal MMP-9 in 33 patients with IPAA, 23 patients with a PDAI < 7 and 10 patients with a PDAI ≥ 7 and later in 138 patients with inflammatory bowel disease, including 34 patients with IPAA.^37,49^ The median fecal MMP-9 level was higher in the 17 patients diagnosed with pouchitis compared to 17 patients with normal pouches. At a cutoff a fecal MMP-9 level of 0.24 ng/mL, the authors reported an AUC of 0.76, translating to a sensitivity and specificity of 87% for active pouchitis, defined an endoscopic sub-score of the PDAI ≥ 3 (Supplementary Table 6).

In the previously mentioned study by Werner et al., the authors also investigated the association of fecal anti-GP2 and associations with pouchitis.^21^ Fecal samples were obtained in 11 patients with recurrent, acute pouchitis (patients with episodes of antibiotic-responsive pouchitis with repeated flares up to four times per year), 17 with chronic pouchitis (patients requiring antibiotic or anti-inflammatory therapy for at least four weeks or patients having more than five flares per year), nine with a normal pouch, and five with FAP. Patients with a PDAI ≥ 7 had a significantly higher titer of anti-GP2 IgG and IgA compared to patients with a PDAI < 7.

Fecal pyruvate kinase has also been found to correlate with pouchitis in two studies of 81 total patients.^50,51^ The dimeric form of pyruvate kinase is present in polymorphonuclear neutrophils and in other rapidly proliferating cells, especially in the context of environmental stress.^52, 53^ Walkowiak et al. and Johnson et al. both found higher fecal pyruvate concentrations in those with pouch inflammation, though sensitivity/specificity was only calculated in the Johnson study (Supplementary Table 6).

WGLF evaluation is a process in which patients are provided with a colonic prep solution to facilitate the passage of fecal effluent. Previous studies have suggested that immunoglobulins in this fluid remain stable over time, making it ideal for the collection of proteins.^54, 55^ In an initial study by Stallmach et al., IgG, albumin, and soluble CD44 isoforms were evaluated in WGLF in a cohort of 34 patients with IPAA, and there were significant positive correlations between these markers and the endoscopic PDAI subscore (Supplementary Table 7).^56^ Evgenikos et al. subsequently conducted several studies looking at WGLF of various potential markers, including A1AT, granulocyte elastase, Interleukin (IL) 1β, IL 8, and IgG.^57–59^ The sensitivities and specificities of these various WGLF proteins are detailed in Supplementary Table 7. None of the studies utilizing WGLF reported AUCs.

### Phenotypic Discrimination of Pouch Inflammation

None of the studied stool or serum biomarkers have been shown to accurately discriminate between different disease entities of the pouch, including cuffitis, acute pouchitis, recurrent pouchitis, chronic pouchitis, or Crohn’s-like disease of the pouch. Though still limited, the best biomarker for phenotypic discrimination appears to be glycoprotein 2. Werner et al. did find a significant difference in the sera and stool concentration of glycoprotein 2 autoantibodies in patients with chronic pouchitis and recurrent acute pouchitis compared to patients with a normal pouch or in healthy relatives.^60^ In addition, a subset of chronic pouchitis patients with Crohn’s-like disease complications—strictures, fistulae, and pre-pouch inflammation, had significantly higher sera and stool titers of glycoprotein 2 compared to patients with chronic pouchitis without these complications.

### Longitudinal Studies

The majority of the studies included in this systematic review are cross-sectional studies, reporting the result of a biomarker following a singular assessment. The description of longitudinal assessments of biomarkers is reported in only a minority of studies.^6, 7, 19, 47^ Notably, only stool and not serum biomarkers have been studied longitudinally. Ollech et al. predominantly reported the results of a cross-sectional study, but they did describe the repeated assessment of fecal calprotectin in 50 patients.^19^ However, neither the results of these longitudinal assessments nor the changes in biomarkers following medical interventions were detailed.^19^ In contrast, Boerr et al. describe 52 determinations of fecal A1AT in a cohort of 33 patients. In a subset of 16 patients with pouchitis, treatment with a three-week course of metronidazole was described, with appropriate reduction in stool excretion of fecal A1AT in follow-up; however, the exact data from these assessments are not reported.^47^ Similarly, in a subset of seven patients, Gonsalves et al. reported an improvement in lactoferrin level that correlated with clinical improvement as assessed by the clinical PDAI following a course of antibiotics. The lactoferrin level correlated with the PDAI scores and was able to determine the presence or resolution of inflammation post-therapy. Importantly, repeat endoscopic evaluation was not obtained in these patients.^43^ Finally, Werner et al. did not suggest significant changes in autoantibodies targeting glycoprotein 2 in a subset of patients who were followed longitudinally.^61^

## Discussion

IPAA is the primary surgical option for the management of medical refractory UC and complications associated with UC. Despite significant improvements in quality-of-life following IPAA creation, pouch inflammation with associated symptoms of frequency, urgency, rectal bleeding, incontinence, and abdominal pain occurs in the majority of patients post-operatively.^62, 63^ Nevertheless, alternative diagnoses can cause similar symptoms. A noninvasive, cost-effective, and objective means of differentiating inflammatory pathologies from non-inflammatory pathologies, as well as monitoring for improvement or worsening of disease, is of vital importance.

An ideal biomarker—one that is easily measurable, noninvasive, inexpensive, rapidly accessible, reproducible, and of sufficiently high sensitivity and specificity to discriminate between diseased and non-diseased states—could transform the care of patients with IPAA.

In this systematic review, we thus sought to evaluate all serum, stool, urine, and breath biomarkers utilized in the evaluation of pouch inflammation in comparison to the gold standard of pouchoscopy. While a variety of serum and stool biomarkers were investigated, our search strategy identified no studies with validated biomarkers in urine and breath in comparison to pouch endoscopy.

Serum biomarkers represent an appealing option for the evaluation of pouch inflammation. Considering the relatively noninvasive collection process, a blood-based biomarker for the assessment of pouchitis would be ideal. However, the compiled studies indicate that current biomarkers in use have significant limitations. CRP and A1AT have inadequate sensitivity and specificity. Other serum tests, such as serum serotonin, although potentially attractive, lack standardization. Finally, the compiled data on the use of p-ANCA in pouch inflammation demonstrates conflicting results. The current serum-based evaluations of inflammation may increase suspicion for the presence of ongoing inflammatory burden, but would likely merit the use of additional, often invasive, testing for confirmation in clinical practice.

Either in comparison to other biomarkers or unilaterally against the gold standard of pouchoscopy, fecal calprotectin is the most commonly studied biomarker for the evaluation of pouch inflammation. Nevertheless, there are clear limitations to the evidence presented. All studies presented are observational, with concerns related to selection bias. The heterogeneity in study design, especially as it relates to the inclusion of patients with diagnoses other than UC with IPAA, adds an additional level of confounding. Moreover, the commonly used modalities for assessing fecal calprotectin reported in the study were ELISA and quantitative immunochromatography, and correlations between these tests can vary by as much as 50%.^64^ ELISA testing also has a significant amount of inter-test variability.^65, 66^ Given these limitations along with the variation in cutoff values and wide-ranging sensitivities and specificities, there are significant barriers to standardizing the use of fecal calprotectin for the assessment of pouch disease.

Other stool biomarkers for the assessment of pouch inflammation include fecal lactoferrin, fecal A1AT, fecal MMP-9, and fecal pyruvate kinase. Of these markers, fecal lactoferrin demonstrated the most consistently high sensitivities and specificities.^42, 43, 67^ The authors of two of these studies suggest the use of fecal lactoferrin algorithmically in the evaluation of pouch inflammation to exclude irritable pouch syndrome.^42,67^ An additional cost-effectiveness study by Parsi et al. suggested improved cost with initial testing of fecal lactoferrin prior to the administration of antibiotics.

Older studies by Evengenikos and Stallmach evaluated the role of WGLF.^56,58,68,69^ Although these studies suggested varying sensitivities and specificities, the evaluations are likely limited in the clinical setting by the requirement of bowel preparation and associated patient discomfort.

Overall, there are a number of important limitations to this systematic review. The biggest limitation is the quality of evidence surrounding biomarkers. As already outlined, no randomized control trials were available. Only two studies reported the predictive value of biomarkers for the evaluation of pouch inflammation following IPAA creation. Moreover, a minority of studies provided longitudinal assessments of pouch inflammation against the gold standard of pouchoscopy. Only a single study suggested discriminatory behavior in terms of phenotypic subtypes of pouch inflammation.^21^ In addition, comparisons across studies are limited due to varying definitions of pouch inflammation, different patient populations, and different biomarker assays. Owing to this heterogeneity of available data, a meta-analysis was unable to be performed. Finally, it is important to note that although pouchoscopy remains the gold standard, significant inter-rater variability exists even in the endoscopic evaluation of the pouch.^70^ As such, much of the variability in sensitivities and specificities reported in the current systematic review could also be attributed to the lack of a truly validated standard for comparison.^71^

In conclusion, unfortunately, no serum or stool biomarker is able to qualify as an ideal marker of pouch inflammation. A clear unmet need exists with respect to the assessment of pouch inflammation. The ability to use biomarkers to risk-stratify patients and provide prognostic assessments is also limited. Controlled studies evaluating biomarkers, not only in serum and stool but potentially attainable through other readily available sources, such as urine and breath, are warranted.

## Supplementary Material

otac043_suppl_Supplementary_Tables

## Data Availability

No new data was created or analyzed.
