# Supplementary material for: Biomarkers for the Evaluation of Pouch Inflammation: A Systematic Review
Source: Crohns Colitis 360. 2022 Nov 24;4(4):otac043. doi: 10.1093/crocol/otac043 (PMC9802421; doi:10.1093/crocol/otac043)
Supplement: otac043_suppl_Supplementary_Tables [file otac043_suppl_supplementary_tables.docx]

**Supplementary Figure 1. PRSIMA Diagram of Included Studies**


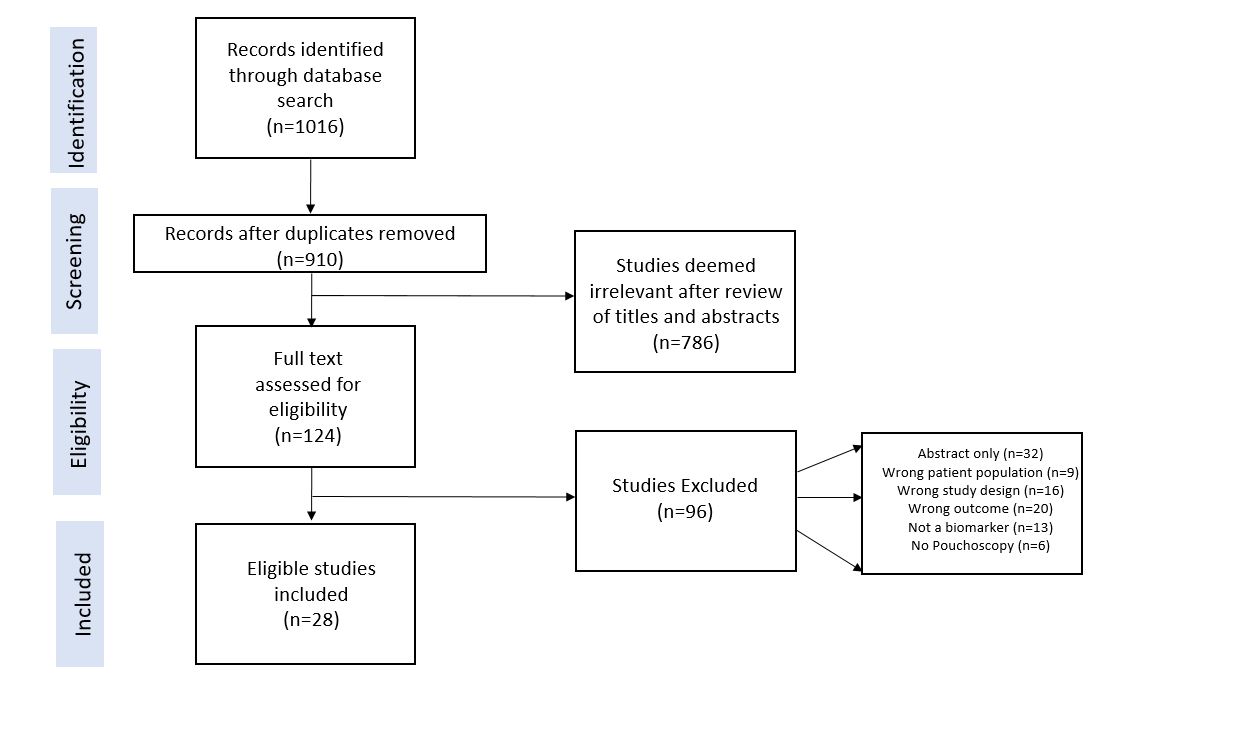


Figure Legend: PRISMA flowchart outlining literature search

**Supplementary Table 1: Ovid MEDLINE Search Terms and Results for Biomarkers and Pouchitis**

Ovid MEDLINE(R) and Epub Ahead of Print, In-Process & Other Non-Indexed Citations and Dally <1946 to February 22, 2022>

| **#** | **Searches** | **Results** | **Type** |
| --- | --- | --- | --- |
| 1 | exp Diagnosis/ | 91242489 | Advanced |
| 2 | (diagnos* or prognos* or predict* or detect* or monitor* or assess* or risk).mp. | 12640731 | Advanced |
| 3 | 1 or 2 | 16276268 | Advanced |
| 4 | Colonic Pouches/ | 1805 | Advanced |
| 5 | Pouchitis/ | 927 | Advanced |
| 6 | (((ileoanal or J or colonic) adj3 pouch*) or pouchitis).mp. | 4031 | Advanced |
| 7 | or/4-6 | 4031 | Advanced |
| 8 | exp Biomarkers/ | 833579 | Advanced |
| 9 | (((serum or serolog*) adj3 (marker* or biomarker*)) or "c reactive protein" or "alpha 1 antitrypsin" or "matrix metalloproteinase" or MMP or cytokine* or "chemokine IL-8" or ASCA or "Anti saccharomyces cerevisiae antibod*" or ANCA or "Anti neutrophil cytoplasmic antibod*" or ((stool or fecal) adj3 (marker* or biomarker*)) or (fecal and (pyruvate or calprotectin or lactoferrin)) or (breath adj3 (marker* or biomarker*)) or (breath and (pentane or ethane or propane or "1 octene" or "3 methylhexane" or "1 decene" or "nitric oxide")) or ((serum or urine) adj3 headspace)).mp. | 669812 | Advanced |
| 10 | 8 or 9 | 1398140 | Advanced |
| 11 | 3 and 7 and 10 | 161 | Advanced |

**Supplementary Table 2: Embase Search Terms and Results for Biomarkers and Pouchitis**

Embase <1974 to 2022 February 22>

| **#** | **Searches** | **Results** | **Type** |
| --- | --- | --- | --- |
| 1 | exp diagnosis/ | 7207100 | Advanced |
| 2 | (diagnos* or prognos* or predict* or detect* or monitor* or assess* or risk).mp. | 17015069 | Advanced |
| 3 | 1 or 2 | 18370320 | Advanced |
| 4 | ileal pouch-anal anastomosis/ | 1713 | Advanced |
| 5 | ileitis/ | 6350 | Advanced |
| 6 | (((ileoanal or J or colonic) adj3 pouch*) or pouchitis).mp. | 4675 | Advanced |
| 7 | or/4-6 | 373664 | Advanced |
| 8 | biological marker/ | 833579 | Advanced |
| 9 | ((serum or serolog*) adj3 (marker* or biomarker*)) or "c reactive protein" or "alpha 1 antitrypsin" or "matrix metalloproteinase" or MMP or cytokine* or "chemokine IL-8" or ASCA or "Anti saccharomyces cerevisiae antibod*" or ANCA or "Anti neutrophil cytoplasmic antibod*" or ((stool or fecal) adj3 (marker* or biomarker*)) or (fecal and (pyruvate or calprotectin or lactoferrin)) or (breath adj3 (marker* or biomarker*)) or (breath and (pentane or ethane or propane or "1 octene" or "3 methylhexane" or "1 decene" or "nitric oxide")) or ((serum or urine) adj3 headspace)).mp. | 1117112 | Advanced |
| 10 | 8 or 9 | 1427368 | Advanced |
| 11 | 3 and 7 and 10 | 824 | Advanced |

**Supplementary Table 3: Cochrane Search Terms and Results for Biomarkers and Pouchitis**

Cochrane Library Date Run :23/02/2022

| **#** | **Searches** | **Results** | **Type** |
| --- | --- | --- | --- |
| #1 | MeSH descriptor: [Diagnosis] explode all trees | 349614 | Advanced |
| #2 | diagnos* or prognos* or predict* or detect* or monitor* or assess* or risk | 954536 | Advanced |
| #3 | #1 OR #2 | 1071063 | Advanced |
| #4 | MeSH descriptor: [Colonic Pouches] this term only | 53 | Advanced |
| #5 | MeSH descriptor: [Pouchitis] this term only | 42 | Advanced |
| #6 | ((ileoanal OR J OR colonic) NEAR/3 pouch*) OR pouchitis | 340 | Advanced |
| #7 | #4 OR #5 OR #6 | 340 | Advanced |
| #8 | MeSH descriptor: [Biomarkers] explode all trees | 22124 | Advanced |
| #9 | ((serum OR serolog*) NEAR/3 (marker* OR biomarker*)) OR "c reactive protein" OR "alpha 1 antitrypsin" OR "matrix metalloproteinase" OR MMP OR cytokine* OR "chemokine IL-8" OR ASCA OR "Anti saccharomyces cerevisiae antibod*" OR ANCA OR "Anti neutrophil cytoplasmic antibod*" OR ((stool OR fecal) NEAR/3 (marker* OR biomarker*)) OR (fecal AND (pyruvate OR calprotectin OR lactoferrin)) OR (breath NEAR/3 (marker* OR biomarker*)) OR (breath AND (pentane OR ethane OR propane OR "1 octene" OR "3 methylhexane" OR "1 decene" OR "nitric oxide")) OR ((serum or urine) NEAR/3 headspace) | 45821 | Advanced |
| #10 | #8 OR #9 | 63264 | Advanced |
| #11 | #3 AND #7 AND #10 | 30 | Advanced |

**Supplementary Table 4: Final Reference List**

| **Author** | **Year** | **Journal** | Study Type |
| --- | --- | --- | --- |
| Boerr | 1995 | European Journal of Gastroenterology and Hepatology | Case Control |
| Evgenikos | 2000 | European Journal of Gastroenterology and Hepatology | Cross Sectional |
| Evgenikos | 2000 | British Journal of Surgery | Cross Sectional |
| Evgenikos | 2002 | Diseases of the Colon and Rectum | Cross Sectional |
| Farkas | 2014 | Expert Review of Gastroenterology and Hepatology | Cross Sectional |
| Farkas | 2015 | Journal of Crohn’s and Colitis | Cross Sectional |
| Gonsalves | 2013 | Diseases of the Colon and Rectum | Cross Sectional |
| Johnson | 2008 | European Journal of Gastroenterology and Hepatology | Cross Sectional |
| Johnson | 2009 | European Journal of Gastroenterology and Hepatology | Cross Sectional |
| Lim | 2008 | Diseases of the Colon and Rectum | Cross Sectional |
| Lu | 2010 | Inflammatory Bowel Diseases | Cross Sectional |
| Machiels | 2017 | Gut | Cross Sectional |
| Matalon | 2015 | Inflammatory Bowel Diseases | Cross Sectional |
| Nishida | 2020 | PLOS ONE | Cross Sectional |
| Ollech | 2021 | Clinical Gastroenterology and Hepatology | Cross Sectional |
| Pakarinen | 2010 | Inflammatory Bowel Diseases | Cross Sectional |
| Parsi | 2004 | Gastroenterology | Cross Sectional |
| Pronio | 2016 | Rev Esp Enfer Diag | Cross Sectional |
| Sandborn | 1995 | American Journal of Gastroenterology | Cross Sectional |
| Scarpa | 2011 | Surgery | Cross Sectional |
| Stallmach | 1999 | International Journal of Colorectal Disease | Cross Sectional |
| Stallmach | 2000 | Gut | Case Control |
| Thomas | 2000 | Diseases of the Colon and Rectum | Cross Sectional |
| Walkowiak | 2005 | Scandinavian Journal of Gastroenterology | Cross Sectional |
| Wang | 2013 | Journal of Crohn’s and Colitis | Cross Sectional |
| Werner | 2013 | Journal of Crohn’s and Colitis | Case Control |
| Yanamato | 2015 | American Journal of Gastroenterology | Case Control |
| Yasuda | 1998 | Scandinavian Journal of Gastroenterology | Cross Sectional |

**Supplementary Table 5: Risk of Bias Assessment – Newcastle Ottawa Scale**

| **Author** | **Year** | **Selection Subscore (Max 4)** | **Comparability Subscore (Max 2)** | **Exposure Subscore (Max 3)** | **Outcome Subsocre (Max 3)** | **Total Score (Max 9)** |
| --- | --- | --- | --- | --- | --- | --- |
| Boerr | 1995 | 2 | 2 | 3 | 3 | 7 |
| Evgenikos | 2000 | 3 | 2 | 3 | NA | 8 |
| Evgenikos | 2000 | 3 | 2 | 3 | NA | 8 |
| Evgenikos | 2002 | 3 | 2 | 3 | NA | 8 |
| Farkas | 2014 | 3 | 2 | 3 | NA | 8 |
| Farkas | 2015 | 3 | 2 | 3 | NA | 8 |
| Gonsalves | 2013 | 3 | 2 | 3 | NA | 8 |
| Johnson | 2008 | 2 | 2 | 3 | NA | 7 |
| Johnson | 2009 | 2 | 2 | 3 | NA | 7 |
| Lim | 2008 | 3 | 2 | 3 | NA | 8 |
| Lu | 2010 | 3 | 2 | 3 | NA | 8 |
| Machiels | 2017 | 3 | 2 | 3 | NA | 7 |
| Matalon | 2015 | 2 | 2 | 2 | NA | 6 |
| Nishida | 2020 | 3 | 2 | 2 | NA | 7 |
| Ollech | 2021 | 2 | 2 | 3 | NA | 7 |
| Pakarinen | 2010 | 3 | 2 | 3 | NA | 8 |
| Parsi | 2004 | 3 | 2 | 3 | NA | 8 |
| Pronio | 2016 | 2 | 2 | 3 | NA | 8 |
| Sandborn | 1995 | 1 | 2 | 3 | NA | 6 |
| Scarpa | 2011 | 3 | 2 | NA | 3 | 8 |
| Stallmach | 1999 | 2 | 2 | 3 | NA | 7 |
| Stallmach | 2000 | 2 | 2 | 3 | 3 | 7 |
| Thomas | 2000 | 2 | 2 | 2 | NA | 6 |
| Walkowiak | 2005 | 2 | 2 | 3 | NA | 7 |
| Wang | 2013 | 3 | 2 | 3 | NA | 8 |
| Werner | 2013 | 2 | 2 | 2 | NA | 6 |
| Yanamato | 2015 | 2 | 2 | 3 | NA | 7 |
| Yasuda | 1998 | 1 | 2 | 3 | NA | 6 |

**Supplementary Table 6: Exploratory Stool Markers**

| **Author** | **Year** | **Study Design** | **Patient #** | **Median Age (range)** | **Pre-op Diagnosis** | **Pouchitis Definition** | **Biomarker** | **Cut-Off** | **Association Between Biomarker and PDAI** | **Sensitivity (%)** | **Specificity (%)** |
| --- | --- | --- | --- | --- | --- | --- | --- | --- | --- | --- | --- |
| Boerr | 1995 | Case Control | 33  (52 samples) | 37-41 (21-68) | UC | Pouchitis: PDAI >7  Remission: symptoms resolved by time of study with PDAI <6 | Fecal A1AT | 20.5ng | Positive Correlation between PDAI and Fecal Alpha-1 Anti-trypsin  r=0.702 p<0.0001 | 80 | 97 |
| Parsi | 2004 | Prospective Cohort | 49 | 42-53 (36-57) | UC | PDAI ≥7 | Fecal A1AT | NP | Fecal A1AT unable to distinguish inflammatory pouch disease from non-inflammatory pouch disease  AUC: 0.377  No correlation between with PDAI:  r=0.86 (-0.10-0.47), p=0.20 | NP | NP |
| Walkowiak | 2005 | Prospective Cohort | 27 | 38  (18-59) | UC  (18)  FAP  (9) | PDAI ≥7  Moskowitz Criteria ≥5 | Fecal Pyruvate Kinase  (M2-PK) | NP | Higher fecal pyruvate kinase in IPAA with a PDAI > 7 or Moskowitz > 5 | NP | NP |
| Johnson | 2009 | Prospective Cohort | 54 | 48 | UC  (46)  FAP  (8) | PDAI ≥7 | Fecal Pyruvate Kinase  (M2-PK) | ≥4 U/mL | dM2-PK concentrations correlated well with PDAI  r=0.64  P < 0.0001 | 73.9  80 | 70.6  71 |
| Farkas | 2014 | Prospective Cohort | 33 | 30-40 | UC | PDAI ≥7 | Fecal MMP-9 | 3.86 ng/mL | Fecal MMP-9 concentration and severity of pouchitis correlated  r=0.526 p=0.017  AUC: 0.89 | 89 | 91 |
| Farkas | 2015 | Prospective Cohort | 138 | 38-43 | 50 CD  54 UC  34 IPAA (for UC) | PDAI ≥7 | Fecal MMP-9 | 0.24 ng/mL | Fecal MMP-9 correlated with endoscopic activity  p=0.011  AUC: 0.76 | 87 | 87 |

Table 6 Legend: pre-op = preoperative, UC = ulcerative colitis, CD = Crohn’s disease, IPAA = ileal pouch-anal anastomosis, PDAI = pouchitis disease activity index, A1AT = alpha 1 anti-trypsin, MMP = matrix metalloprotease, AUC = area under the curve, IPAA=ileal pouch anal anastomosis

**Supplementary Table 7: Association of WGLF Proteins with Pouch Inflammation**

| **Author** | **Year** | **Study Design** | **Patient #** | **Median Age (range)** | **Pre-Op Diagnosis** | **Pouchitis Definition** | **Biomarker** | **Cut-Off** | **Association Between Biomarker and PDAI** | **Sensitivity (%)** | **Specificity (%)** |
| --- | --- | --- | --- | --- | --- | --- | --- | --- | --- | --- | --- |
| Evgenikos | 2000 | Prospective Cohort | 56 | 43 | UC | Moskowitz Criteria | WGLF GE | Detectable (39 nkat / L) | Significantly higher WGLF GE (p<0.001) | 71 | 81 |
| Evgenikos | 2000 | Prospective Cohort | 42 | 43 | UC | Moskowitz Criteria  PDAI ≥7 | WGLF IgG    WGLF Albumin  WGLF A1AT | >10 μg/mL  26 μg/mL  NA | Significantly higher WGLF IgG (p<0.001)  Significantly higher WGLF IgG (p<0.001)  No correlation | 71  80 | 91  97 |
| Evgenikos | 2002 | Prospective Cohort | 42 | 39 (20-73) | UC | Moskowitz Criteria  PDAI ≥7 | WGLF IL8  WGLF IgG  WGLF GE | >200 ng/mL  >10 μg/mL  > 39 nkat / L | Significantly higher WGLF proteins | 100  80  60 | 86  97  84 |
| Stallmach | 1999 | Prospective Cohort | 34 | NP | UC (25)  FAP (8) | PDAI ≥7 | WGLF IgG  WGLF Albumin  WGLF sCD44 | 10 mg/l 30 mg/l  30 mg/l 90 mg/l  0.5 μg/l 2 μg/l | Correlation between PDAI and WGLF proteins (r=0.672 to 0.769) | 88 75  88 75  88 75 | 77 96  58 96  82 100 |

Table 7 Legend: Pre-op = pre-operative, PDAI = pouchitis disease activity index; WGLF = Whole gut lavage fluid; GE = Granulocyte Elastase; IgG = immunoglobulin G, IL = interleukin, scD44 = soluble cluster of differentiation 44
